# Aromatic, Sensory, and Fatty Acid Profiles of Arbequina Extra Virgin Olive Oils Produced Using Different Malaxation Conditions

**DOI:** 10.3390/foods11213446

**Published:** 2022-10-30

**Authors:** Alexandra Olmo-Cunillera, Enrico Casadei, Enrico Valli, Julián Lozano-Castellón, Eleftherios Miliarakis, Inés Domínguez-López, Antònia Ninot, Agustí Romero-Aroca, Rosa Maria Lamuela-Raventós, Maria Pérez, Anna Vallverdú-Queralt, Alessandra Bendini

**Affiliations:** 1 Department of Nutrition, Food Science and Gastronomy, XIA, Faculty of Pharmacy and Food Sciences, Institute of Nutrition and Food Safety (INSA-UB), University of Barcelona, 08028 Barcelona, Spain; 2 CIBER Physiopathology of Obesity and Nutrition, Institute of Health Carlos III, 28029 Madrid, Spain; 3 Department of Agricultural and Food Sciences, Alma Mater Studiorum—Università di Bologna, 47521 Cesena, Italy; 4 Interdepartmental Centre for Industrial Agrofood Research, Alma Mater Studiorum—Università di Bologna, 47521 Cesena, Italy; 5 IRTA Institute of Agrifood Research and Technology, Fruit Science Program, Olive Growing and Oil Technology Research Team, 43120 Constantí, Spain; 6 Laboratory of Organic Chemistry, Faculty of Pharmacy and Food Sciences, University of Barcelona, 08028 Barcelona, Spain

**Keywords:** organoleptic, fruity, bitter, hexanal, oleic acid, multivariate analysis

## Abstract

The demand for high-quality extra virgin olive oil (EVOO) is growing due to its unique characteristics. The aroma and flavor of EVOO depend on its content of volatile organic compounds (VOCs), whose formation is affected by the olive variety and maturity index, and the oil production process. In this study, the sensory quality and VOC and fatty acid (FA) profiles were determined in Arbequina olive oils produced by applying different malaxation parameters (20, 25, and 30 °C, and 30 and 45 min). All the olive oils were classified as EVOO by a sensory panel, regardless of the production conditions. However, cold extraction at 20 °C resulted in more positive sensory attributes (complexity). The FA concentration increased significantly with the malaxation temperature, although the percentage profile remained unaltered. Finally, an OPLS-DA model was generated to identify the discriminating variables that separated the samples according to the malaxation temperature. In conclusion, the tested range of malaxation parameters appeared not to degrade the distinctive attributes/organoleptic profile of olive oil and could be applied to obtain an EVOO of high sensory quality, especially at 20 °C.

## 1. Introduction

Extra virgin olive oil (EVOO) is highly appreciated for its distinctive aroma and flavor, as well as its multiple health benefits [1]. EVOO consists mainly of triglycerides (TAG) and a variety of minor compounds, including volatile organic compounds (VOCs), free fatty acids (FAs), phenolic compounds, tocopherols, pigments, sterols, waxes, and hydrocarbons [2]. The nutritional and health-promoting properties of EVOO are mainly correlated with its highly bioactive components, such as monounsaturated FAs (MUFAs), unsaponifiable compounds, and soluble or hydrophilic compounds, including *α*-tocopherol, phenolic compounds, and other antioxidants [3]. 

A high proportion of the glyceride fraction of EVOO consists of FAs, particularly MUFAs (55–83% of the oil), which have only one double bond in their structure. This feature makes EVOO more resistant to oxidation and contributes to its antioxidant properties and long shelf life compared with oils rich in polyunsaturated FAs (PUFA) [4]. Typically, the unsaturated FAs in EVOO form up to 80–85% of the oil composition, with the contents of oleic (C18:1, 55–83% of total FA), linoleic (C18:2, 3.5–21%), and palmitoleic (C16:1, 0.3–3.5%) acids being notably high. In contrast, saturated FAs constitute only about 14% of EVOO, mainly consisting of palmitic (C16:0, 7.5–20%) and stearic (C18:0, 0.5–5%) acids [5].

When FAs undergo specific reactions, mainly catalyzed by lipoxygenases (LOX), but also involving autoxidation or photooxidation mechanisms, they produce VOCs, low-molecular-weight components that volatilize at room temperature [6] and generate the organoleptic profile of olive oils [7]. VOCs are classified as ketones, ethers, esters, aldehydes, alcohols, and hydrocarbons, among others [8]. Given that VOCs are responsible for both positive and negative olfactory attributes [9], they play a key role in oil quality and consumer preferences. 

The aromatic profile of olive oil is affected by several factors, including the cultivar, olive ripening stage, environmental growing conditions, and processing and storage conditions [10,11,12]. These factors contribute to the great variety and complexity of olive oil flavors. Reactions catalyzed by endogenous enzymes generate the VOCs responsible for the EVOO aromas perceived as positive [7]. For example, the typical fruity and green sensory notes arise from the large amounts of C5 and C6 VOCs (alcohols, aldehydes, and esters) generated through the LOX pathway [6,13]. On the other hand, unpleasant aromatic compounds are generally formed by the chemical oxidation of the oil and exogenous enzymes [14]. Phenolic compounds also contribute to the sensory quality of EVOO, being responsible for bitterness, astringency, and pungency [15]. 

In recent years, high-quality EVOOs (also known as premium EVOOs) have become increasingly available on the market. Although not an official category established by regulations, EVOOs are recognized as high quality if they have outstanding organoleptic characteristics [16]. These properties can only be achieved with strictly controlled production conditions, such as harvesting the olives at a green stage when lipogenesis is incomplete and the fat component is lower. Another factor is the use of a cold extraction process, in which EVOOs are produced at a temperature below 27 °C [17] or even below 20 °C to avoid the volatilization of VOCs [18]. Malaxation at temperatures above 30 °C leads to the loss of aromas and enhances oxidation, but oil yields are higher, which may be of interest to some producers [19]. However, oil mills aiming to produce high-quality EVOOs need to apply lower temperatures.

The effect of malaxation conditions on EVOO quality and composition has been extensively studied, but different conditions and olive varieties to those of the present work were used. For example, Angerosa et al. [20] processed oils of the Italian Coratina and Frantoio varieties at 25 and 35 °C for 15, 30, 45, 60, and 90 min. Taticchi et al. [21] studied the influence of three temperatures (20, 25, and 35 °C) on oils of the Coratina, Ogliarola, Moraiolo. and Peranzana varieties without considering the time of malaxation, whereas Marx et al. [22] investigated the effect of malaxation at three temperatures (22, 28, and 34 °C) for 60 min on oils obtained from Cobrançosa olives. Arbequina oils have also been studied, as here, but with a different experimental design and using olives from other regions. For example, using olives picked in Córdoba, Spain, Vidal et al. [19] evaluated the effect of the malaxation temperature and time (values not specified) together with the ripening index (RI) (from 0 to 3) and an irrigated or rainfed crop to ascertain which conditions yielded oils with more VOCs and pigments. In another study, Arbequina olives (RI = 2) picked in Huelva, Spain, were used to produce EVOO with malaxation at 30 °C for 45 min [23].

In the present work, different times (30 and 45 min) and temperatures (20, 25, and 30 °C) of malaxation were applied to produce Arbequina EVOO on a laboratory scale. Arbequina is the main olive variety cultivated and used for EVOO production in the region of Catalonia, where the study was performed [24]. The main goal was to evaluate the sensory quality of the EVOOs obtained and the effect of cold extraction (carried out at 20 and 25 °C). Three aspects were studied: (i) the FA composition, which is related to some of the health properties of EVOO; (ii) the qualitative and quantitative profiles of the EVOO volatile fraction, which contribute to the flavor and aroma of the oil; and (iii) the sensory attributes of the EVOOs, which were analyzed by a professional panel to verify if any differences could be perceived.

Additional relevant information is provided regarding the quality of the EVOO samples analyzed in a previous study [25], which aimed to evaluate the effect of the aforementioned malaxation conditions on the bioactive components of oil. 

## 2. Materials and Methods

### 2.1. Reagents

*n*-Hexane, 0.5 N sodium methoxide, and 14% boron trifluoride–methanol were purchased from Sigma-Aldrich (St. Louis, MO, USA); sodium chloride (NaCl) from Panreac Química SLU (Castellar del Vallès, Spain); and anhydrous sodium sulfate (Na_2_SO_4_) for gas chromatography (GC) from Scharlau (Sentmenat, Spain). Ultrapure water was obtained using a Milli-Q purification system (Millipore, Bedford, MA, USA). 

The tridecanoic acid (C13:0) methyl ester was used as a standard for the analysis of FAs and was acquired from Sigma-Aldrich. The following standards (CAS number and purity percentage in parenthesis) were used for the analysis of VOCs and were purchased from Sigma-Aldrich: (*E*)-2-decenal (3913-81-3, ≥95.0%), (*E*)-2-heptenal (18829-55-5, ≥95%), (*E*)-2-hexenal (6728-26-3, ≥97.0%), (*E*,*E*)-2,4-hexadienal (142-83-6, ≥95.0%), (*Z*)-3-hexenyl acetate (3681-71-8, ≥98.0%), 1-hexanol (111-27-3, ≥99.9%), 1-octen-3-ol (3391-86-4, ≥98.0%), 3-methyl-1- butanol (123-51-3, ≥98.5%), 6-methyl-5-hepten-2-one (110-93-0, ≥97.0%), acetic acid (64-19-7, ≥99.8%), ethanol (64-17-5, ≥99.9%), ethyl acetate (141-78-6, ≥99.8%), ethyl propanoate (105-37-3, ≥99.7%), hexanal (66-25-1, 98%), nonanal (124-19-6, ≥95%), octane (111-65-9, ≥99.7%), pentanoic acid (109-52-4, ≥99.8%), and propanoic acid (79-09-4, ≥99.8%). 4-Methyl-2-pentanol (123-51-3, ≥95%) was used as an internal standard.

### 2.2. Samples

The olive oil samples used were the same as those used in the study by Olmo-Cunillera et al. [25]. They consisted of six Arbequina EVOOs produced by an Abencor system using different temperatures (20, 25, and 30 °C) and times (30 and 45 min) of malaxation. The quality parameters (acidity, peroxide value, and specific extinctions in UV) of the EVOOs and the characteristics of the olives used are described in Olmo-Cunillera et al. [25]. The olives were harvested during the second week of November 2019, and their RI ranged from 1.16 to 2.26.

### 2.3. Extraction and Determination of FAs

FAs were extracted following the method for FA methyl esters (FAME) described in López-López et al. [26]. An amount of 25 mg of olive oil was weighed in a 10 mL tube and 100 µL of the internal standard (tridecanoic acid, C13) was added at 400 ppm. Firstly, after the addition of 2 mL of 0.5 N sodium methoxide, the solution was stirred for 30 s and immediately heated at 100 °C for 15 min. The samples were then cooled in an ice bath. Secondly, 2 mL of 14% boron trifluoride was added to the samples, and the solution was again stirred for 30 s and heated at 100 °C for 15 min before cooling in an ice bath. Thirdly, 1 mL of hexane was added to the samples and the solution was stirred for 30 s. After the incorporation of 2 mL of saturated NaCl, the samples were stirred again for 30 s. Finally, the samples were centrifuged at 3000 rpm for 7 min, and the hexane phase was collected in an Eppendorf tube containing anhydrous Na_2_SO_4_, mixed, and left to stand for 5 min. The liquid was then collected with a micropipette and stored in vials at −20 °C until analysis. 

Fast GC analyses were performed on a Shimadzu GC-2010 Gas Chromatograph (Shimadzu, Kyoto, Japan) equipped with a flame ionization detector and a Shimadzu AOC-20i Autoinjector. Separation of FAME was carried out on a capillary column (40 cm × 0.18 mm i.d. × 0.1 µm film thickness) coated with an RTX-2330 stationary phase of 10% cyanopropyl phenyl–90% biscyanopropyl polysiloxane from Restek (Bellefonte, USA). 

The operating conditions were as follows: the split/splitness injector was used in the split mode with a split ratio of 1:50; the injection volume of the sample was 1 µL; and the injector and detector temperatures were kept at 250 °C and 300 °C, respectively. The temperature program was as follows: initial temperature 110 °C, increased at 52 °C/min to 195 °C and held at this temperature for 6 min, and then increased at 25 °C/min until 230 °C and held for 6.5 min (total run time: 16.03 min). Hydrogen was used as the carrier gas at a constant pressure of 26 psi, referring to a linear velocity of 40 cm/s at 110 °C. Data acquisition and processing were performed with Shimadzu-Chemstation software for GC systems.

The concentration of every FA was calculated considering the area and concentration of the internal standard, applying the following equation:

(A_i_ × C_IS_) / (A_IS_ × M_S_),

where A_i_ is the area of the FA, C_IS_ is the concentration of the internal standard, A_IS_ is the area of the internal standard, and M_S_ is the mass of the sample. The percentage composition was calculated by dividing the area of the FA by the sum of the area of all identified FAs and multiplying by 100.

### 2.4. Extraction and Determination of VOCs

The procedures to prepare the internal standard solution and samples were described in Casadei et al. [27] and Aparicio-Ruiz et al. [28]. The sample, placed in a 20 mL vial closed with a septum (polytetrafluoroethylene), was left for 10 min at 40 °C under agitation to allow for the equilibration of the volatiles in the headspace (HS). After that, the solid-phase microextraction (SPME) fiber was exposed to the HS for 40 min at 40 °C, which was carried out with the assistance of an autosampler (AOC-5000 plus, Shimadzu, Kyoto, Japan). The fiber was then inserted into the injector port of the GC for 5 min at 250 °C with the purge valve off (splitless mode) and injected into a polar-phase capillary column (TG-WAXMS: length 60 m, internal diameter 0.25 mm, and coating 0.50 µm; Thermo Fisher Scientific, Waltham, MA, USA) of a GC with a mass spectrometry (MS) detector (QP2010 Ultra, Shimadzu, Kyoto, Japan). The ion source and transfer line temperature were 200 °C and 260 °C, respectively. The MS analyzer was operated in the full-scan mode (*m/z* range from 30 to 250), with a scan speed of 454 (*m/z*)/s and electron energy of 70 eV. The carrier gas used was helium, and the oven temperature was held at 40 °C for 10 min and then programmed to increase by 3 °C/min to a final temperature of 200 °C. A cleaning step was added at the end of the oven programmed temperature (20 °C/min to 250 °C for 5 min) to ensure that the column was ready for the next analysis.

### 2.5. Sensory Analysis

The sensory analysis was performed by the Official Tasting Panel of Catalonia according to regulations of the European Union (UE 2568/91, update) [29] and IOC (IOC/T.20 Doc. No. 15/Rev. 10/2018) [30]. 

The different oil samples were sensorily profiled according to the intensity of defects and three main positive attributes (fruity, bitterness, and pungency), as determined by a group of tasters selected, trained, and monitored as a panel. As described in the official method (IOC/T.20/Doc. No. 15/Rev. 10/2018) [30], each taster wrote down the perceived intensity of every negative and positive attribute on a 10 cm scale, where 0 cm means the absence of the attribute and 10 cm represents the maximum intensity of a given attribute. The taster could move between both edges to decide the perceived intensity for a particular sample. The Official Tasting Panel of Catalonia is trained in the use of this intensity scale, as required for recognition by the IOC and EU. Finally, for every descriptor, the median score of the eight tasters of the panel was computed and given as the intensity. 

Additionally, secondary positive attributes described in IOC rules were used: astringent, grass, green, apple, sweet, banana, etc. The overall sensory perceptions were graded using a similar continuous scale, determining the complexity of sensations. For complexity, the panel evaluated the combination of the different positive sensations perceived for each olive oil. A higher number of perceived sensations resulted in greater complexity. The Panel of Catalonia is officially recognized by the EU and IOC and follows ISO 17025 rules. Samples were presented randomly to the eight trained tasters of the panel on the same day, grouped into tasting sessions of four samples with ten-minute breaks between sessions.

### 2.6. Statistical and Multivariate Analyses

All malaxation treatments, as well as the FA and VOC determination, were carried out in triplicate. An analysis of variance (ANOVA) with a Tukey test was performed to assess the effect of the malaxation temperature and time on the FA composition using STATGRAPHICS Centurion 18 software, version 18.1.13 (Statgraphics Technologies, INC, The Plains, Virginia). The results relating to the VOCs underwent statistical analysis. XLSTAT software (Addinsoft Corp., Paris, France) was used to perform the ANOVA, selecting a Brown–Forsythe test.

Additionally, multivariate analysis was performed with all the data collected in the present study plus the previously published data on phenolic content [25]. Phenolic compounds were grouped by classes (secoiridoids, lignans, phenolic acids, phenolic alcohols, and flavonoids), and only oleocanthal and oleacein were included individually. The software used was SIMCA 13.0.3.0 (Umetrics, Umeå, Sweden). First, an unsupervised approach, specifically a principal component analysis (PCA), showed that the samples could be separated by their malaxation temperature. Then, supervised analysis, specifically the orthogonal projections to latent structures–discriminant analysis (OPLS-DA) model, was conducted in order to find the discriminating variables that separated the EVOO samples according to their malaxation temperature. The EVOO samples were distributed on the X-axis according to the malaxation temperature (20, 25, and 30 °C). OPLS-DA was chosen because orthogonal variability was dominant in X (orthogonal *R^2^X* = 0.520 vs predictive *R^2^X* = 0.244) [31]. This indicated that only 24.4% of the variation in the EVOO samples correlated with the temperature of malaxation and that most of the variation correlated with other variables. The model had two predictive components and five orthogonal components, and accounted for 76.4% of the X-variation (*R^2^X*) and 98.5% of the Y-variation (*R^2^Y*). The quality and reliability of the model were assessed by the following parameters. *R^2^Y* (explained variation) was 0.985, which referred to the goodness of fit (how well the data of the training set can be mathematically reproduced) and *Q^2^* (predicted variation) was 0.971, which referred to the predictive power of the model. Additionally, to assess the reliability of the OPLS-DA model, a cross-validated ANOVA (CV-ANOVA) was performed, and a *p*-value of <0.01 was obtained, indicating that it was a significant model. The permutation test (200 permutations) was carried out to exclude overfitting. Hotelling’s T2 and DModX were performed to identify strong and moderate outliers, and none were found. Furthermore, variable importance in the projection (VIP) values of >1 were accepted as the most influential for the model and compared with their coefficient values. Coefficient values of >1 and <1 express how strongly variables are positively and negatively correlated with the X classes (temperature of malaxation), respectively, as long as their confidence interval does not include zero. 

## 3. Results and Discussion

### 3.1. Determination of FAs

Table 1 and Table 2 show the concentration and percentage, respectively, of the FAs detected in our EVOO samples, which were principally oleic acid (C18:1 n-9) (68–71%), followed by palmitic acid (C16:0) (14–16%) and linoleic acid (C18:2 n-6) (9–11%). Stearic (C18:0) and palmitoleic (C16:1 n-7) acids accounted for 1.5–2% and 1–1.5% of the FAs, respectively, and the others had proportions below 1%. The FA composition (%) of the samples (Table 2) fell within the limits established for EVOO by the European Union (UE 2568/91, update) [29]: myristic (C14:0) ≤ 0.03%, *α*-linolenic (C18:3 n-3) ≤ 1.00%, arachidic (C20:0) ≤ 0.60%, gondoic (eicosenoic) (C20:1 n-9) ≤ 0.50%, behenic (C22:0) ≤ 0.20%, and lignoceric (C24:0) ≤ 0.20%. The following FAs also complied with the regulation: palmitic (C16:0) 7.50–20.00%, palmitoleic (C16:1 n-7) 0.30–3.50%, margaric (heptadecanoic) (C17:0) ≤ 0.40%, heptadecenoic (C17:1) ≤ 0.60%, stearic (C18:0) 0.50–5.00%, oleic (C18:1 n-9) 55.00–83.00%, and linoleic (C18:2 n-6) 2.50–21.00%. Only the myristic acid content in samples produced at 30 °C was slightly higher than the required 0.03% (0.04%); this is not the first time that an Arbequina EVOO had a higher level of this FA [32,33].

The results showed that the FA concentration (Table 1) was significantly lower in oils produced at 20 °C, and the lowest concentration was found after malaxation at 20 °C for 30 min. As the olives used to produce the EVOOs shared the same characteristics, it can be assumed that the conditions of the malaxation process were responsible for this variation. During malaxation, the solid and liquid phases are separated, generating an oily phase that contains TAGs, other non-polar compounds (sterols, waxes, hydrocarbons, and pigments), emulsified polar compounds (mainly water), and small solid particles. As the oil droplets merge, a process that increases with malaxation time and temperature, the oily phase increases in TAGs, as well as other non-polar compounds. In contrast, the emulsified polar compounds transfer to the water phase, and the small solid particles to the solid phase. Therefore, efficient separation of the oily phase requires a suitable adjustment of malaxation parameters. Higher temperatures during malaxation (up to 30 °C) reduce viscosity and enhance the coalescence of oil droplets, leading to higher yields [34], which could explain why TAGs, and consequently FAs, increased with the malaxation temperature. As the temperature increased, the oily phase became richer in oil and poorer in the other compounds, especially unsaponifiable lipids and water. Among the tested conditions, malaxation at 20 °C for 30 min was the least effective for separating the oily phase, resulting in an oil with a lower TAG and FA concentration. 

Although the malaxation time did not have a significant effect on the FA content in general, it seems that, at lower temperatures (20 °C) (Table 1), longer malaxation times (45 min) could favor the coalescence of oil droplets and a proper separation of the oily phase, and thus produce EVOOs with higher TAG and FA concentrations. In contrast, at a higher temperature (30 °C), extending the malaxation time could have a negative effect on the TAG content due to oxidation, although, in our study, the quality parameters (K_232_ ≤ 2.50, K_270_ ≤ 0.22, ∆K ≤ 0.01, peroxide value ≤ 20 mEq O_2_/kg, and acidity ≤ 0.8 g oleic acid/100 g) indicated that the tested temperatures did not induce a significant oxidation process [25]. It was, therefore, demonstrated that a malaxation temperature of 30 °C was not high enough to oxidize and damage the lipid fraction of the EVOO within the studied time periods. 

Regardless of these changes, the percentages of individual FAs remained the same (Table 2) as in previous studies [35,36,37,38], indicating that the FA profile of these EVOOs was maintained within malaxation parameters of 20, 25, and 30 °C and 30 and 45 min.

It is worth noting that the EVOOs produced at 25 °C for 45 min and at 30 °C for 30 min had a higher percentage of stearic, oleic, *α*-linolenic (C18:3 n-3), and arachidic acid (C20:0), and a lower percentage palmitic and palmitoleic acid (Table 2). Palmitic acid has negative health associations, as it is known to contribute to cardiovascular diseases [39], whereas oleic and *α*-linoleic acids have cardiovascular protective effects [39]. Although these variations found might be insufficient to cause any health effects, the data could be of interest for future studies on the effect of malaxation conditions on the FA content in EVOO.

### 3.2. Determination of the Volatile Fraction

The EVOO samples analyzed in this study were obtained from olives in a good state of conservation, resulting in an aromatic fraction mainly composed of C5 and C6 compounds derived from primary and secondary LOX pathways, which are associated with positive sensory attributes (Table S1). C6 compounds are produced by endogenous enzymes that use linolenic and α-linolenic acids as initial substrates [40]. This process generates a wide variety of VOCs, which are responsible for the sensory profile of high-quality EVOOs appreciated by consumers [41]. The ripening stage of olives is a crucial parameter in the formation of VOCs through the LOX pathway, with the enzymatic activity decreasing as the fruit matures. During the initial phase of inolition (maturation phase in which the lipid content of the fruit increases), olives contain practically equal quantities of C6 aldehydes and C6 alcohols. Almost all C6 aldehydes reach their maximum concentration in the subsequent veraison stage (maturation phase in which the color of the fruit epicarp changes) [42]. 

The amount of VOCs determined in EVOO depends partly on the methodology used. In the present work, to obtain the VOC profile of the EVOO samples (Table 3), HS-SPME analysis was performed. Aldehydes were the principal class of identified and quantified molecules, followed by alcohols and ketones; esters, pentene dimers, hydrocarbon structures, and terpenes exhibited lower concentrations. The aroma of EVOO is attributed to aldehydes, alcohols, esters, ketones, terpenes, and hydrocarbons [42]. The principle C6 compounds identified were (*E*)-2-hexenal, (*Z*)-2-hexenal, hexanal, (*E*)-3-hexen-1-ol, hexyl acetate, (*Z*)-3-hexen-1-ol acetate, and (*E*)-2-hexen-1-ol (Table 3). All of them, except for the latter, varied significantly in concentration according to the malaxation time–temperature binomial. As the EVOO samples were all produced from the same olive variety using fruit with a very similar RI at the time of harvest, their enzymatic patrimony was uniform and typical of oils produced from yellow–green olives. However, the activity of individual LOX enzymes can be influenced by malaxation conditions [41]. 

In addition to C6 compounds, positive sensory attributes are imputable to C5 compounds generated by a secondary branch of the LOX pathway [43]. Except for the C6 aldehydes derived from linolenic acid (ƩC6 LnA-Ald, sum of (*E*)-2-hexenal and (*Z*)-2-hexenal), among which (*E*)-2-hexenal is the main component, no major differences in concentration were detected between C5 and C6 compounds in the analyzed samples, with their levels ranging from 0.1 to 1 ppm (Table S1). In contrast, in other studies with Arbequina olive oil, the amount of C6 compounds was found to be 2- to 160-fold higher than that of other chemical classes of volatile molecules [44]. Nevertheless, irrespective of their concentration in the HS, VOCs are crucial in determining the quality of virgin olive oil [7]. 

The increase in malaxation time from 30 to 45 min at 20 and 25 °C significantly increased the amount of (*E*)-2-hexenal and (*Z*)-3-hexen-1-ol acetate, whereas, at 30 °C, the highest content of these VOCs was obtained after kneading the olive paste for only 30 min, probably because they evaporate over time.

The predominant VOCs in most EVOOs are C6 aldehydes, such as hexanal, responsible for the aromas of green apple and cut grass, and (*E*)-2-hexenal, associated with bitter almond, green fruit, sharp, bitter, and astringent notes. Among the C6 alcohols, hexan-1-ol imparts tomato, fruity, aromatic, soft, alcoholic, and rough aromas [7,41,45]. Reboredo-Rodríguez et al. [46] observed a clear difference in the odorant series of EVOOs (Morisca and Manzanilla de Sevilla) produced with either 30 min or 90 min of malaxation at 30 °C. In the Morisca oil, the total concentration of C6 volatiles decreased when the paste was processed at 30 °C instead of 20 °C, regardless of the kneading time. On the other hand, when the malaxation time was reduced from 90 min to 30 min, an increase in the total amount of C5 compounds was observed.

In a previous study, Angerosa et al. [20] concluded that a shorter malaxation time (between 30 and 45 min) at a low temperature (25 °C) was optimal for the processing of Coratina and Frantoio olive paste in terms of VOCs associated with pleasant sensory notes. When the tests were performed at 35 °C with prolonged times (more than 45 min), there was a marked decrease in C6 esters and (*Z*)-3-hexen-1-ol, both strong contributors to green aroma, as well as an increase in hexan-1-ol and (*E*)-2-hexen-1-ol, considered responsible for less attractive sensory perceptions.

The most abundant C5 compounds derived from the LOX pathway were found to be pentene dimers, and six isomeric structures of these molecules were tentatively identified and grouped. Cavalli et al. [47] reported that pentene dimers, along with a low amount of C5 ketones, positively influence the aroma of olive oil. Among the C5 ketones detected in the present study, 1-penten-3-one was found in all the samples, its concentration tending to increase with the malaxation temperature. When the temperature was raised by 10 °C (from 20 to 30 °C), the concentration almost doubled (Table 3).

### 3.3. Sensory Analysis

**Figure 1:**
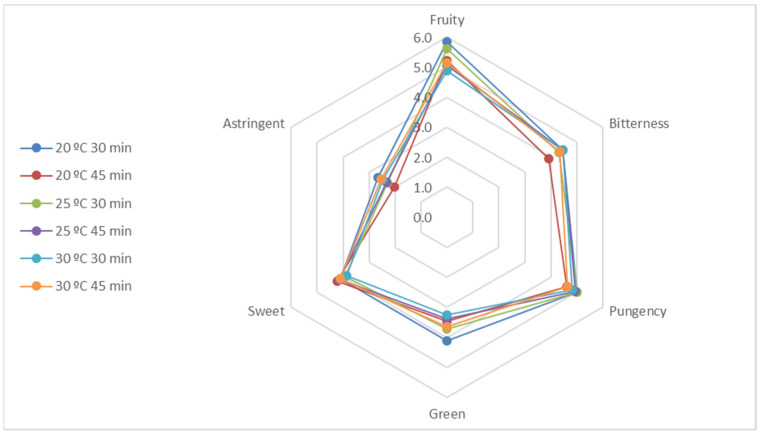
Sensory evaluation of the Arbequina EVOO samples produced using a specific temperature (20, 25, and 30 °C) and time (30 and 45 min) of malaxation. The attributes represented are fruity, bitterness, pungency, green, sweet, and astringent. The scores are given on a 10 cm scale.

The Official Tasting Panel of Catalonia characterized all olive oil samples as belonging to the EVOO category in accordance with the quality parameters [25]. The perception of sensory defects (fusty/muddy sediment, musty–humid–earthy, winey–vinegary–acid–sour, frostbitten olives, and rancid) and other negative attributes was null, whereas the perception intensity of the three main positive attributes (fruity, bitterness, and pungency) was high for all EVOO samples (Figure 1), especially for the EVOO produced at 20 °C for 30 min, which had the highest fruity values. This agrees with the VOC results and the association of high-quality EVOO with a fruitier attribute. Moreover, aromatic notes such as green, sweet, almond, apple, banana, tomato plant, grass, leaves, fennel, and artichoke were noticeable in all samples. According to the literature, these notes can be related to some of the VOCs found, such as ethanol (apple and sweet), pentan-1-ol (fruity and pungent), 2-penten-1-ol (almond, banana, fruity, grass, and green), (*E*)-2-hexen-1-ol (apple, fruity, grass, green, leaves, and sweet), hexanal (apple, banana, grass, green, and sweet), (*E*)-2-hexenal (almond, apple, astringent, bitter, fruity, green, leaves, and sweet), and 1-penten-3-one (bitter, green, pungent, sweet, and tomato) [48]. 

The fruity note is characteristic of oil produced from healthy, fresh olives and is mainly associated with pentanol, hexanol, butyl acetate, and hexyl acetate. The green attribute is characteristic of unripe olives and is produced by (*E*)-2-pentenal, hexanal, (*Z*)-3-hexenal, (*E*)-2-hexenal, (*E*)-2-hexen-1-ol, and (*E*)-3-hexen-1-ol. Bitter notes are characteristic of olive oil obtained from green olives or those beginning to ripen and are correlated with 1-penten-3-one, although the main contributors are phenolic compounds. 1-penten-3-one, together with some phenolic compounds, are also positively correlated with pungency, which refers to the biting tactile sensation characteristic of oils produced at the start of the crop year, primarily from unripe olives; (*E*)-2-hexenal and hexanal are negatively correlated with pungency [7]. Finally, astringency is attributed to phenolic compounds, such as flavonoids and oleacein [49].

Other studies have found that bitterness and pungency are less perceptible after malaxation at temperatures above 35 °C due to the considerable reduction in secoiridoids and 1-penten-3-one and the inactivation of enzymes; moreover, the levels of compounds contributing to positive sensory characteristics of EVOO remain high after processing for between 30 and 45 min [20,22,38,50]. In contrast, other studies describe an increase in bitterness and pungency with malaxation temperature, corresponding to an increase in phenolic compounds [49]. Additionally, Boselli et al. [50] concluded that the sensory quality of EVOOs produced at temperatures below 27 °C or at 35 °C was similar. In our study, the increases in pungency, bitterness, and astringency with temperature were in accordance with the increase in the secoiridoid oleocanthal [25]. 

Nonetheless, the chemical reactions that take place in the malaxer are highly diverse, with significant interactions between the numerous compounds involved. In addition, a compound may be synthesized without migrating to the oily phase intact, as partition reactions are complex. All these factors could explain the divergent results among studies.

### 3.4. Multivariate Analyses by OPLS-DA

**Figure 2:**
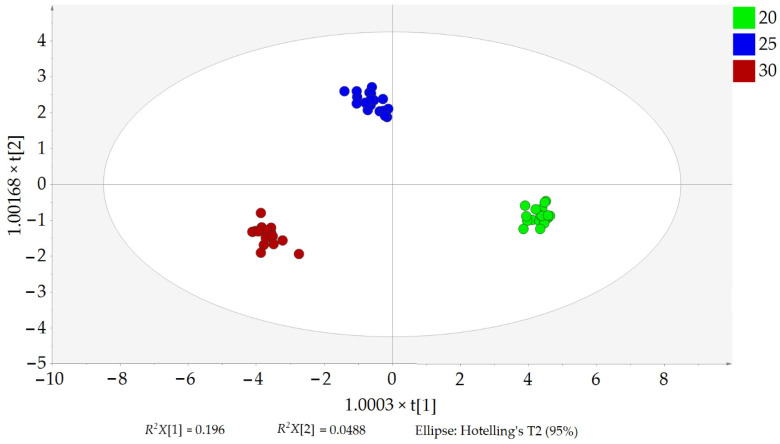
Score scatter plot of the OPLS-DA. EVOO samples are colored according to the malaxation temperature (20, 25, and 30 °C). *R^2^X*[1] and *R^2^X*[2] are the values with variation in the two predictive components based on the malaxation temperature. Their sum is *R^2^X* = 0.244, which refers to the variation correlated with the malaxation temperature. All EVOO samples were inside the Ellipse Hotelling’s T2, meaning that there were no strong outliers.

Once the OPLS-DA was performed, the EVOO samples were clearly grouped into three clusters according to their malaxation temperature on the X-axis (20, 25, and 30 °C; from right to left) (Figure 2).

**Figure 3:**
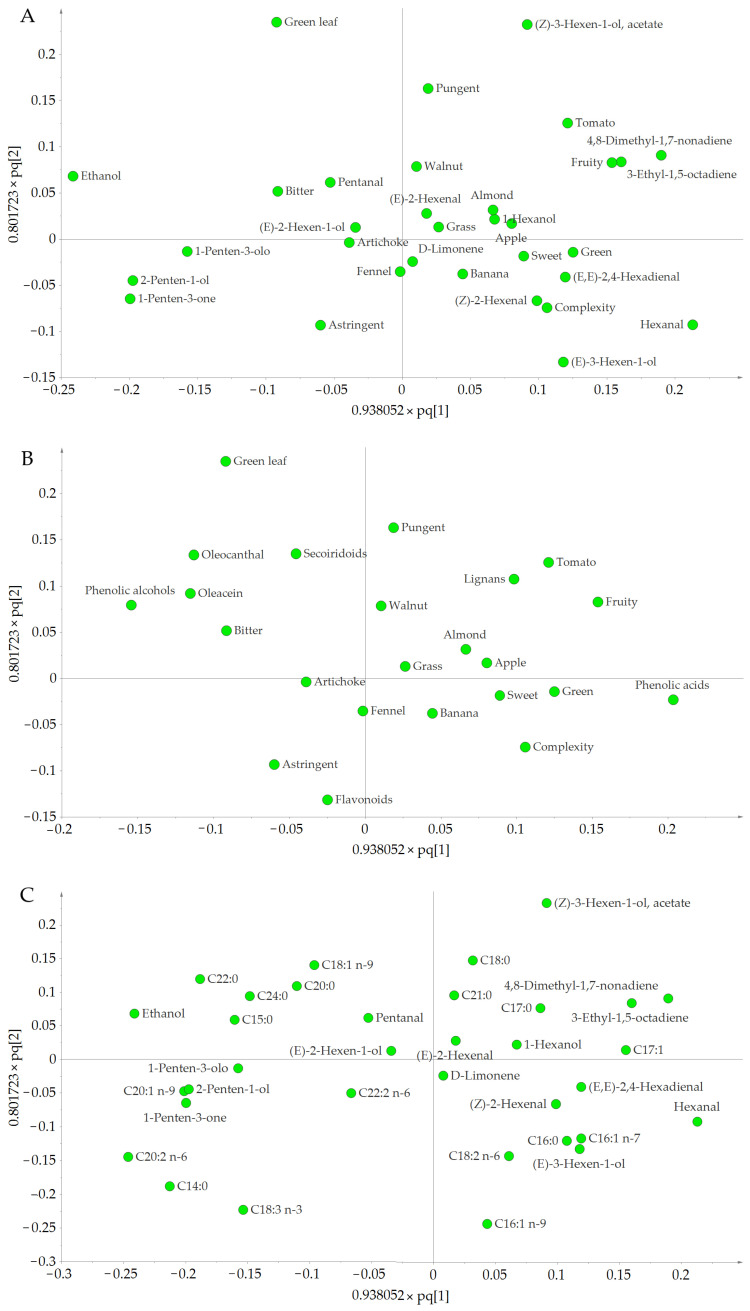
Loading scatter plots of the OPLS-DA showing the distribution and correlation of the different parameters analyzed in the EVOO samples. (**A**) Distribution and correlation of the VOCs and sensory characteristics. (**B**) Distribution and correlation of the phenolic compounds and sensory characteristics. (**C**) Distribution and correlation of the VOCs and FAs.

The loading plot (Figure 3) shows the characteristics of the samples according to the analyzed variables, as well as their correlations. The variables located in the bottom right were characteristic of EVOO samples produced at 20 °C, in the upper middle at 25 °C, and lower left at 30 °C. These results were verified by the coefficient values. Several observations can be made from these data.

Regarding the sensory attributes, it was found that EVOOs produced at 20 °C were positively and significantly correlated with the aromatic notes of sweet and apple (Figure 3A). The complexity (number of perceived sensations) was also positively and significantly correlated with temperature (the higher the complexity, the more aromatic descriptors a particular EVOO had). Thus, EVOOs produced at 20 °C were the most complex in terms of sensory characteristics. This is in accordance with the association of high-quality EVOOs with production by cold extraction. Similarly, EVOOs produced at 25 °C were positively and significantly correlated with certain aromatic notes, above all, green leaf, but also tomato, fruity, and grass, as well as pungency. However, they were negatively correlated with complexity. In contrast, EVOO samples produced at 30 °C had significant negative correlations with most of the aromatic notes (green leaf, tomato, sweet, apple, and grass), fruity, and complexity, but were positively correlated with banana and fennel, as well as astringency and bitterness. This indicates that sensory attributes related to aromatic notes start to disappear when the temperature increases, as it promotes the evaporation of VOCs, which are responsible for the aromatic characteristics of oil, and the inactivation of hydroperoxide lyase [51]. Nevertheless, certain aromatic descriptors seemed to be highly produced when malaxating at a particular temperature, such as green leaf and tomato at 25 °C, apple at 20 °C, and banana and fennel at 30 °C.

A similar scatter plot distribution could be observed for the VOCs. Most of the molecules, especially the C6 compounds, were located on the right (Figure 3A). Thus, VOCs such as hexanal, (*E*)-3-hexen-1-ol, 3-ethyl-1,5-octadiene, 4,8-dimethyl-1,7-nonadiene, (*E*)-2-hexen-1-ol, and (*E*,*E*)-2,4-hexadienal were positively and significantly correlated with EVOOs produced at 20 °C, whereas VOCs such as (*Z*)-3-hexen-1-ol acetate, ethanol, and 1-hexanol were characteristic of EVOOs produced at 25 °C. The C5 group (2-penten-1-ol, 1-penten-3-one, and 1-penten-3-ol) was mostly found on the left, and was positively and significantly correlated with 30 °C, indicating a higher content in EVOOs produced at this temperature. (*E*)-3-hexen-1-ol and D-limonene were also positively correlated with 30 °C. In addition, hexanal was strongly correlated with 20 °C, indicating that it was formed at low temperatures. This agrees with the results of Salas et al. [51], who found that the maximum formation of hexanal occurred at 15 °C. Hexanal is the precursor of hexanol, which was only positively correlated with 25 °C, suggesting that this temperature might be favorable for the enzymatic activity involved in its transformation [52].

Analysis of VOCs and sensory attributes revealed the following associations. VOCs such as hexanal, (*E*)-3-hexen-1-ol, 3-ethyl-1,5-octadiene, 4,8-dimethyl-1,7-nonadiene, (*E*)-2-hexen-1-ol, and (*E*,*E*)-2,4-hexadienal were more likely to contribute to sweet and apple notes, as well as to the complexity of the aromatic descriptors, which was characteristic of EVOOs produced at 20 °C. VOCs such as (*Z*)-3-hexen-1-ol acetate, ethanol, and 1-hexanol were more likely to contribute to tomato, green leaf, and fruity aromatic notes characteristic of EVOOs produced at 25 °C. Finally, 2-penten-1-ol, 1-penten-3-one, and 1-penten-3-ol were more likely to contribute to the banana note, astringency, and bitterness attributes characteristic of EVOOs produced at 30 °C. Although the correlations between VOCs and aromatic notes are complex, as the concentration of each VOC needs to be equal to or higher than its threshold value to be detected by the olfactory receptors [53] and more than one VOC can contribute to the same aromatic note, our results coincide reasonably with those in the literature [7,48,52]. Regarding D-limonene, it could be related to the fennel aromatic note, as they were closely situated in the loading plot (Figure 3A), and both positively correlated with 30 °C. Moreover, other studies report that this VOC contributes to the aroma of fennel oil [54].

The coefficient values of the sensory attributes and phenolic compounds (Figure 3B) showed that, at 20 °C pungency and bitterness were negatively and significantly correlated, as were oleocanthal and oleacein. In contrast, at 25 °C, a significant positive correlation was found for pungency and oleocanthal and a significant negative correlation for flavonoids and astringency; bitterness and oleacein were not significant. Finally, the characteristic sensory attributes at 30 °C were astringency and bitterness, and a significant positive correlation was found for flavonoids and oleacein. According to these results, flavonoids are more likely to make a higher contribution to astringency, oleocanthal to pungency, and oleacein to bitterness, which agrees with the aforementioned studies [49,55]. 

The distribution of the most important FAs and their coefficient values revealed that EVOOs produced at 30 °C were richer in *α*-linolenic, gondoic, and linoleic acids; at 25 °C, in lignoceric, behenic, oleic, and stearic acids; and at 20 °C, in palmitoleic, palmitic, *α*-linolenic, and linoleic acids (Figure 3C). These results match the analysis of FA profiles, which found a higher percentage of palmitic and palmitoleic acids in EVOOs produced at 20 °C compared with 25 and 30 °C.

The variables that most influenced the OPLS-DA model (VIP > 1) and could be used to discriminate between the three temperature clusters were, in descending order, C14:0, C18:3 n-3, C20:2 n-6, C16:1 n-9, green leaf, (*Z*)-3-hexen-1-ol acetate, ethanol, hexanal, C22:0, 4,8-dimethyl-1,7-nonadiene, 1-penten-3-one, C20:1 n-9, phenolic acids, (*E*)-3-hexen-1-ol, 2-penten-1-ol, tomato, 3-ethyl-1,5-octadiene, pungent, C18:1 n-9, oleocanthal, C16:1 n-7, phenolic alcohols, fruity, C24:0, and C18:2 n-6. According to their coefficients, C18:3 n-3, C16:1 n-9, hexanal, 4,8-dimethyl-1,7-nonadiene, phenolic acids, (E)-3-hexen-1-ol, 3-ethyl-1,5-octadiene, and C16:1 n-7 were characteristic of EVOOs produced at 20 °C; green leaf, (Z)-3-hexen-1-ol acetate, ethanol, C22:0, tomato, pungent, C18:1 n-9, oleocanthal, fruity, and C24:0 at 25 °C; and C14:0, C18:3 n-3, C20:2 n-6, 1-penten-3-one, C20:1 n-9, 2-penten-1-ol, (E)-3-hexen-1-ol, and C18:2 n-6 at 30 °C. Considering these results, a good marker of Arbequina EVOOs produced at 20 °C could be high levels of hexanal, as well as the content of palmitoleic acid (C16:1), whereas 1-penten-3-one and 2-penten-1-ol could be markers of Arbequina EVOOs produced at 30 °C.

Finally, it should be noted that the OPLS-DA model explained only 24.4% of the variation in the EVOO samples in correlation with the temperature of malaxation. Therefore, a significant part of the variation was associated with other variables (orthogonal variability *R^2^X* = 52%), which could include the malaxation time and the RI of the olives.

## 4. Conclusions

The sensory characteristics of EVOOs are related to different constituents, above all, VOCs. The content of VOCs in the EVOO, and therefore its sensory quality, depends on the processing conditions, among other factors. High-quality EVOOs are produced at temperatures below 27 °C and claim to have an extraordinary quality. This study demonstrated that varying the malaxation parameters between 20 and 30 °C and 30 and 45 min induced differences in the sensory attributes of Arbequina EVOO. Although the Official Tasting Panel perceived aromatic notes in almost all of the samples, EVOOs malaxed at 20 °C for 30 min were the fruitiest. Furthermore, the OPLS-DA model was able to discriminate between sensory characteristics according to the malaxation temperature. For example, EVOOs produced at 20 °C had more apple and sweet notes, and a greater complexity of sensory descriptors than the others, whereas those produced at 25 °C had more green leaf, tomato, and fruity notes and pungency, and those produced at 30 °C had more banana and fennel notes, as well as astringency and bitterness.

The positive qualities detected by the Official Tasting Panel were associated with the identified VOCs. The malaxation time and temperature significantly affected the levels of some VOCs, and the OPLS-DA model showed that high levels of hexanal were characteristic of EVOOs produced at 20 °C, whereas 1-penten-3-one and 2-penten-1-ol were associated with those produced at 30 °C.

The FA profile (composition percentages) was maintained regardless of the malaxation time and temperature. However, the FA concentrations increased at 30 °C, suggesting that higher temperatures improved the separation of the oil phase, resulting in a higher concentration of TAGs. The OPLS-DA model provided similar information, as some FAs had VIP values of >1, indicating their importance for the projection of the model, and they could be used to discriminate between the three clusters of temperature. Five of them (C14:0, C18:3 n-3, C20:2 n-6, C20:1 n-9, and C18:2 n-6) were positively and significantly correlated with 30 °C. Strikingly, EVOOs malaxed at 25 °C for 45 min and at 30 °C for 30 min had a lower content of palmitic acid and higher levels of oleic and *α*-linolenic acid, which could be of interest for future studies given the negative health effects of palmitic acid. However, unlike oleic and *α*-linolenic acids, palmitic acid did not have a VIP of >1 in the OPLS-DA model, indicating a low contribution to the separation of the clusters according to malaxation temperature, which would, therefore, not greatly affect its content.

Finally, when also considering the phenolic content of these EVOO samples (obtained in a previous study), it could be concluded that, although all the malaxation times and temperatures tested here resulted in Arbequina EVOOs of high sensory quality, cold extraction at 20 °C provided extra quality. These findings indicate that the processing conditions can be varied within the studied parameters without negative effects on sensory characteristics or the EVOO status of the final product, whereas producers seeking extra quality value can select a malaxation temperature of 20 °C and time of 30 min.

## Figures and Tables

**Table 1:** Concentration (mg/g) of the fatty acids identified in the EVOO samples.

Fatty Acid	Concentration (mg/g oil) ^1^					
	20 °C		25 °C		30 °C	
	30 min	45 min	30 min	45 min	30 min	45 min
C14:0	0.20 ± 0.02 ^a^	0.23 ± 0.01 ^ab^	0.29 ± 0.02 ^c^	0.26 ± 0.01 ^bc^	0.35 ± 0.02 ^d^	0.33 ± 0.03 ^d^
C15:0	0.08 ± 0.01 ^a^	0.10 ± 0.01 ^ab^	0.15 ± 0.04 ^bc^	0.12 ± 0.01 ^bc^	0.13 ± 0.00 ^c^	0.14 ± 0.01 ^c^
C16:0	110.63 ± 8.06 ^a^	128.46 ± 12.13 ^b^	146.65 ± 4.52 ^c^	135.72 ± 9.13 ^bc^	136.15 ± 5.25 ^bc^	140.67 ± 3.83 ^bc^
C16:1 n-9	0.98 ± 0.08 ^a^	1.06 ± 0.11 ^ab^	1.17 ± 0.04 ^bc^	1.22 ± 0.06 ^c^	1.27 ± 0.04 ^c^	1.18 ± 0.03 ^bc^
C16:1 n-7	10.29 ± 0.89 ^ab^	12.49 ± 1.31 ^cd^	13.59 ± 0.41 ^d^	9.96 ± 0.68 ^a^	10.22 ± 0.40 ^a^	11.59 ± 0.31 ^bc^
C17:0	1.22 ± 0.09 ^a^	1.20 ± 0.14 ^a^	1.31 ± 0.06 ^a^	1.61 ± 0.06 ^b^	1.60 ± 0.05 ^b^	1.27 ± 0.03 ^a^
C17:1	2.29 ± 0.15 ^a^	2.36 ± 0.26 ^ab^	2.56 ± 0.10 ^b^	2.84 ± 0.14 ^c^	2.87 ± 0.10 ^c^	2.43 ± 0.06 ^ab^
C18:0	13.37 ± 0.87 ^a^	14.20 ± 1.30 ^ab^	16.34 ± 0.52 ^c^	18.39 ± 1.07 ^d^	18.26 ± 0.67 ^d^	15.68 ± 0.39 ^bc^
C18:1 n-9	483.05 ± 32.57 ^a^	544.61 ± 49.99 ^b^	626.59 ± 18.21 ^c^	660.03 ± 42.51 ^c^	657.27 ± 24.65 ^c^	615.12 ± 15.78 ^c^
C18:2 n-6	73.78 ± 5.18 ^a^	87.38 ± 8.15 ^b^	100.92 ± 2.93 ^c^	84.34 ± 5.68 ^b^	86.99 ± 3.32 ^b^	96.63 ± 2.51 ^c^
C18:3 n-3	3.97 ± 0.25 ^a^	4.27 ± 0.42 ^a^	4.82 ± 0.16 ^b^	5.28 ± 0.27 ^c^	5.41 ± 0.19 ^c^	5.31 ± 0.14 ^c^
C20:0	2.68 ± 0.19 ^a^	2.97 ± 0.27 ^a^	3.47 ± 0.11 ^b^	3.67 ± 0.21 ^b^	3.70 ± 0.14 ^b^	3.44 ± 0.08 ^b^
C20:1 n-9	1.88 ± 0.15 ^a^	2.24 ± 0.20 ^b^	2.59 ± 0.09 ^c^	2.58 ± 0.16 ^c^	2.64 ± 0.10 ^c^	2.56 ± 0.05 ^c^
C20:2 n-6	0.13 ± 0.01 ^a^	0.15 ± 0.02 ^a^	0.19 ± 0.01 ^b^	0.20 ± 0.01 ^b^	0.26 ± 0.02 ^c^	0.24 ± 0.01 ^c^
C21:0	0.10 ± 0.01 ^a^	0.10 ± 0.01 ^a^	0.13 ± 0.00 ^b^	0.12 ± 0.00 ^b^	0.13 ± 0.01 ^b^	0.12 ± 0.01 ^b^
C22:0	0.80 ± 0.05 ^a^	0.92 ± 0.07 ^b^	1.09 ± 0.07 ^c^	1.11 ± 0.05 ^c^	1.13 ± 0.05 ^c^	1.07 ± 0.02 ^c^
C22:2 n-6	0.19 ± 0.01 ^a^	0.24 ± 0.02 ^b^	0.27 ± 0.02 ^c^	0.26 ± 0.01 ^bc^	0.28 ± 0.01 ^c^	0.27 ± 0.01 ^c^
C24:0	0.48 ± 0.07 ^a^	0.57 ± 0.03 ^b^	0.71 ± 0.06 ^c^	0.69 ± 0.03 ^c^	0.70 ± 0.05 ^c^	0.68 ± 0.02 ^c^
Total FA	706.16 ± 48.48 ^a^	803.63 ± 74.15 ^b^	922.94 ± 27.07 ^c^	928.48 ± 60.01 ^c^	929.40 ± 34.99 ^c^	898.78 ± 23.20 ^c^

^1^ Results are given as the mean ± standard deviation. Three experimental replicates and three analytical replicates were tested for each EVOO sample. Values with the same superscript letters in the same row did not differ significantly between the samples with *p* < 0.05. EVOO, extra virgin olive oil.

**Table 2:** Profile of the fatty acids (%) identified in the EVOO samples.

Fatty Acid	Percentage (%) ^1^					
	20 °C		25 °C		30 °C	
	30 min	45 min	30 min	45 min	30 min	45 min
C14:0	0.03 ± 0.00 ^a^	0.03 ± 0.00 ^a^	0.03 ± 0.00 ^a^	0.03 ± 0.00 ^a^	0.04 ± 0.00 ^b^	0.04 ± 0.00 ^b^
C15:0	0.01 ± 0.00 ^a^	0.01 ± 0.00 ^a^	0.02 ± 0.00 ^b^	0.01 ± 0.00 ^a^	0.01 ± 0.00 ^a^	0.02 ± 0.00 ^b^
C16:0	15.66 ± 0.13 ^b^	15.98 ± 0.12 ^c^	15.89 ± 0.13 ^c^	14.62 ± 0.05 ^a^	14.65 ± 0.02 ^a^	15.65 ± 0.03 ^b^
C16:1 n-9	0.14 ± 0.00 ^a^	0.13 ± 0.01 ^a^	0.13 ± 0.00 ^a^	0.13 ± 0.00 ^a^	0.14 ± 0.00 ^a^	0.13 ± 0.00 ^a^
C16:1 n-7	1.46 ± 0.04 ^c^	1.55 ± 0.05 ^d^	1.47 ± 0.00 ^c^	1.07 ± 0.00 ^a^	1.10 ± 0.00 ^a^	1.29 ± 0.00 ^b^
C17:0	0.17 ± 0.01 ^c^	0.15 ± 0.01 ^b^	0.14 ± 0.00 ^a^	0.17 ± 0.01 ^c^	0.17 ± 0.00 ^c^	0.14 ± 0.00 ^a^
C17:1	0.33 ± 0.01 ^d^	0.29 ± 0.01 ^b^	0.28 ± 0.00 ^a^	0.31 ± 0.01 ^c^	0.31 ± 0.00 ^c^	0.27 ± 0.00 ^a^
C18:0	1.89 ± 0.03 ^c^	1.77 ± 0.00 ^b^	1.77 ± 0.01 ^b^	1.98 ± 0.01 ^e^	1.96 ± 0.01 ^d^	1.74 ± 0.00 ^a^
C18:1 n-9	68.41 ± 0.14 ^b^	67.77 ± 0.23 ^a^	67.89 ± 0.12 ^a^	71.09 ± 0.04 ^d^	70.72 ± 0.02 ^c^	68.44 ± 0.03 ^b^
C18:2 n-6	10.45 ± 0.03 ^c^	10.87 ± 0.03 ^e^	10.94 ± 0.03 ^f^	9.08 ± 0.02 ^a^	9.36 ± 0.02 ^b^	10.75 ± 0.01 ^d^
C18:3 n-3	0.56 ± 0.01 ^b^	0.53 ± 0.01 ^a^	0.52 ± 0.00 ^a^	0.57 ± 0.01 ^b^	0.58 ± 0.00 ^c^	0.59 ± 0.00 ^c^
C20:0	0.38 ± 0.01 ^b^	0.37 ± 0.00 ^a^	0.38 ± 0.00 ^b^	0.40 ± 0.00 ^c^	0.40 ± 0.00 ^c^	0.38 ± 0.00 ^b^
C20:1 n-9	0.27 ± 0.00 ^a^	0.28 ± 0.00 ^b^	0.28 ± 0.00 ^b^	0.28 ± 0.00 ^b^	0.28 ± 0.00 ^b^	0.28 ± 0.00 ^b^
C20:2 n-6	0.02 ± 0.00 ^a^	0.02 ± 0.00 ^a^	0.02 ± 0.00 ^a^	0.02 ± 0.00 ^a^	0.03 ± 0.00 ^b^	0.03 ± 0.00 ^b^
C21:0	0.01 ± 0.00 ^a^	0.01 ± 0.00 ^a^	0.01 ± 0.00 ^a^	0.01 ± 0.00 ^a^	0.01 ± 0.00 ^a^	0.01 ± 0.00 ^a^
C22:0	0.11 ± 0.00 ^a^	0.11 ± 0.00 ^a^	0.12 ± 0.00 ^b^	0.12 ± 0.00 ^b^	0.12 ± 0.00 ^b^	0.12 ± 0.00 ^b^
C22:2 n-6	0.03 ± 0.00 ^a^	0.03 ± 0.00 ^a^	0.03 ± 0.00 ^a^	0.03 ± 0.00 ^a^	0.03 ± 0.00 ^a^	0.03 ± 0.00 ^a^
C24:0	0.07 ± 0.01 ^a^	0.07 ± 0.00 ^a^	0.08 ± 0.00 ^b^	0.07 ± 0.01 ^a^	0.07 ± 0.00 ^a^	0.08 ± 0.00 ^b^

^1^ Results are given as the mean ± standard deviation. Three experimental replicates and three analytical replicates were tested for each EVOO sample. Values with the same superscript letters in the same row did not differ significantly between the samples with *p* < 0.05.

**Table 3:** Concentration (mg/kg) of VOCs identified in the EVOO samples.

Volatile Compound	Concentration (mg/kg) ^1^					
	20 °C		25 °C		30 °C	
	30 min	45 min	30 min	45 min	30 min	45 min
Methanol	0.58 ± 0.06 ^a^	0.75 ± 0.07 ^a^	0.70 ± 0.06 ^a^	0.74 ± 0.13 ^a^	0.81 ± 0.00 ^a^	0.76 ± 0.01 ^a^
Ethanol	0.02 ± 0.00 ^a^	0.03 ± 0.01 ^a^	0.05 ± 0.01 ^b^	0.07 ± 0.00 ^c^	0.07 ± 0.00 ^c^	0.06 ± 0.00 ^b^
3-Ethyl-1,5-octadiene (1-6)	2.26 ± 0.40 ^b^	2.07 ± 0.11 ^b^	1.88 ± 0.09 ^ab^	2.11 ± 0.16 ^b^	1.88 ± 0.40 ^ab^	1.55 ± 0.10 ^a^
Pentanal	0.13 ± 0.02 ^ab^	0.11 ± 0.01 ^ab^	0.11 ± 0.01 ^a^	0.22 ± 0.02 ^b^	0.16 ± 0.01 ^ab^	0.13 ± 0.02 ^ab^
1-Penten-3-one	0.39 ± 0.04 ^ab^	0.42 ± 0.03 ^a^	0.58 ± 0.04 ^abc^	0.57 ± 0.00 ^ab^	0.61 ± 0.02 ^c^	0.62 ± 0.04 ^bc^
4,8-Dimethyl-1,7-nonadien	0.29 ± 0.05 ^c^	0.28 ± 0.02 ^bc^	0.24 ± 0.01 ^abc^	0.28 ± 0.00 ^bc^	0.24 ± 0.05 ^ab^	0.20 ± 0.01 ^a^
Hexanal	0.76 ± 0.08 ^c^	1.00 ± 0.05 ^d^	0.68 ± 0.03 ^bc^	0.38 ± 0.00 ^a^	0.36 ± 0.01 ^a^	0.56 ± 0.02 ^ab^
1-Penten-3-ol	0.18 ± 0.03 ^ab^	0.19 ± 0.03 ^a^	0.23 ± 0.03 ^ab^	0.31 ± 0.00 ^ab^	0.30 ± 0.04 ^b^	0.25 ± 0.02 ^ab^
D-Limonene	0.12 ± 0.02 ^b^	0.11 ± 0.02 ^b^	0.10 ± 0.01 ^b^	0.12 ± 0.00 ^b^	0.16 ± 0.02 ^c^	0.07 ± 0.00 ^a^
(*Z*)-2-Hexenal	0.22 ± 0.03 ^b^	0.22 ± 0.02 ^b^	0.23 ± 0.01 ^b^	0.13 ± 0.01 ^a^	0.11 ± 0.01 ^a^	0.22 ± 0.02 ^b^
(*E*)-2-Hexenal	11.52 ± 1.91 ^a^	17.01 ± 0.89 ^b^	11.46 ± 0.46 ^a^	20.64 ± 3.20 ^b^	18.76 ± 3.29 ^b^	10.99 ± 0.32 ^a^
Hexyl acetate	0.07 ± 0.01 ^bc^	0.05 ± 0.01 ^ab^	0.14 ± 0.01 ^c^	0.10 ± 0.01 ^bc^	0.11 ± 0.00 ^bc^	0.04 ± 0.00 ^a^
(*Z*)-3-Hexen-1-ol, acetate	0.53 ± 0.10 ^c^	0.19 ± 0.03 ^ab^	0.57 ± 0.03 ^c^	0.30 ± 0.05 ^b^	0.26 ± 0.04 ^ab^	0.19 ± 0.01 ^a^
2-Penten-1-ol	0.28 ± 0.05 ^a^	0.27 ± 0.01 ^a^	0.31 ± 0.01 ^a^	0.37 ± 0.00 ^ab^	0.38 ± 0.02 ^b^	0.34 ± 0.00 ^ab^
1-Hexanol	0.40 ± 0.08 ^dc^	0.28 ± 0.02 ^bc^	0.49 ± 0.02 ^d^	0.11 ± 0.01 ^a^	0.10 ± 0.00 ^ab^	0.31 ± 0.02 ^bc^
(*E*)-3-Hexen-1-ol	1.28 ± 0.18 ^c^	1.10 ± 0.05 ^bc^	0.91 ± 0.03 ^bc^	0.30 ± 0.01 ^a^	0.25 ± 0.00 ^ab^	0.94 ± 0.03 ^c^
(*E*)-2-Hexen-1-ol	0.10 ± 0.01 ^a^	0.12 ± 0.01 ^a^	0.11 ± 0.00 ^a^	0.13 ± 0.00 ^a^	0.12 ± 0.02 ^a^	0.09 ± 0.01 ^a^
(*E,E*)-2,4-Hexadienal	0.43 ± 0.06 ^c^	0.33 ± 0.10 ^b^	0.39 ± 0.02 ^bc^	0.21 ± 0.00 ^a^	0.23 ± 0.05 ^a^	0.35 ± 0.01 ^bc^
Acetic acid	0.35 ± 0.05 ^a^	0.34 ± 0.03 ^a^	0.56 ± 0.08 ^b^	0.32 ± 0.01 ^a^	0.48 ± 0.04 ^a^	0.29 ± 0.02 ^a^

^1^ Results are given as the mean ± standard deviation. For each EVOO sample, 3 replicates were tested. Values with the same superscript letters in the same row do not differ significantly between the samples for *p* < 0.05. I.S.: internal standard added for the quantification of the volatile organic compounds (VOCs).

## Data Availability

Data are contained within the article.

## Supplementary Materials

The following supporting information can be downloaded at: https://www.mdpi.com/article/10.3390/foods11213446/s1, Table S1: Volatile compounds C6 and C5 divided into chemical classes and according to their formation from linoleic (LA) and linolenic (LnA) acids.
