# Supplementary material for: Aromatic, Sensory, and Fatty Acid Profiles of Arbequina Extra Virgin Olive Oils Produced Using Different Malaxation Conditions
Source: Foods. 2022 Oct 30;11(21):3446. doi: 10.3390/foods11213446 (PMC9656856; doi:10.3390/foods11213446)
Supplement: Supplementary file 1 [file foods-11-03446-s001.zip › foods-1943721-supplementary.pdf]

Supplementary materials

Table S1. Volatile compounds C6 and C5 divided into chemical classes and according to their formation from linoleic (LA) and linolenic (LnA) acids.

**Fatty acid**	**Concentration (mg/kg) [1]**					
	**20 °C**		**25 °C**		**30 °C**	
	**30 min**	**45 min**	**30 min**	**45 min**	**30 min**	**45 min**
ΣC6 LnA-Ald.[2]	11.73 ± 1.93 [a]	20.51 ± 5.72 [b]	11.69 ± 0.46 [a]	20.76 ± 3.21 [b]	18.90 ± 3.25 [b]	11.21 ± 0.31 [a]
ΣC6 LnA-Alc.[3]	1.38 ± 0.19 [c]	0.94 ± 0.48 [bc]	1.01 ± 0.03 [bc]	0.40 ± 0.05 [a]	0.59 ± 0.41 [ab]	1.04 ± 0.04 [bc]
ΣC6 LA-Ald.[4]	0.76 ± 0.08 [c]	1.00 ± 0.05 [d]	0.68 ± 0.03 [bc]	0.45 ± 0.12 [a]	0.46 ± 0.17 [a]	0.56 ± 0.02 [ab]
ΣC6 LnA-Est.[5]	0.53 ± 0.10 [c]	0.22 ± 0.05 [ab]	0.57 ± 0.03 [c]	0.30 ± 0.05 [b]	0.26 ± 0.04 [ab]	0.19 ± 0.01 [a]
ΣC6 LA-Est.[6]	0.08 ± 0.02 [b]	0.07 ± 0.03 [ab]	0.14 ± 0.01 [c]	0.10 ± 0.01 [bc]	0.08 ± 0.04 [b]	0.04 ± 0.00 [a]
ΣC5 LnA-Alc.[7]	0.49 ± 0.11 [a]	0.46 ± 0.04 [ab]	0.54 ± 0.03 [ab]	0.58 ± 0.17 [a]	0.68 ± 0.06 [b]	0.59 ± 0.01 [ab]
ΣC5 LnA-Ket.[8]	0.46 ± 0.12 [ab]	0.42 ± 0.03 [a]	0.58 ± 0.04 [abc]	0.50 ± 0.12 [ab]	0.70 ± 0.16 [c]	0.62 ± 0.04 [bc]
ΣC5 LnA-Ald.[9]	0.13 ± 0.02 [ab]	0.11 ± 0.01 [ab]	0.11 ± 0.01 [a]	0.18 ± 0.08 [b]	0.14 ± 0.04 [ab]	0.13 ± 0.02 [ab]
Σpen. Dim.[10]	2.26 ± 0.40 [a]	2.07 ± 0.11 [b]	1.88 ± 0.09 [a]	2.11 ± 0.16 [b]	1.88 ± 0.40 [b]	1.55 ± 0.10 [a]

[1] Results are given as "mean ± standard deviation". Values with the same superscript letters in the same row do not differ significantly between the samples for $p < 0.05$. [2] Sum of (*E*)-2-hexenal and (*Z*)-2-hexenal; [3] Sum of (*E*)-3-hexen-1-ol and (*E*)-2-hexen-1-ol; [4] Hexanal; [5] (*Z*)-3-hexen-1-ol-acetate; [6] Hexyl acetate; [7] Sum of 1-penten-3-ol and 2-penten-1-ol; [8] 1-penten-3-one; [9] Pentanal; [10] 3-ethyl-1,5-octadiene (1-6).
